# Comparison of US emergency departments by HIV priority jurisdiction designation: A case for geographically targeted screening in teaching hospitals

**DOI:** 10.1371/journal.pone.0292869

**Published:** 2023-10-18

**Authors:** Christopher L. Bennett, Allan S. Detsky, Carson E. Clay, Janice A. Espinola, Julie Parsonnet, Carlos A. Camargo

**Affiliations:** 1 Department of Emergency Medicine, Stanford University School of Medicine, Stanford, CA, United States of America; 2 Department of Epidemiology, Stanford University School of Medicine, Stanford, CA, United States of America; 3 Institute for Health Policy, Management and Evaluation, Dalla Lana School of Public Health, University of Toronto, Toronto, Ontario, Canada; 4 Department of Medicine, Mount Sinai Hospital and University Health Network, Toronto, Ontario, Canada; 5 Department of Emergency Medicine, Harvard Medical School, Boston, MA, United States of America; 6 Department of Medicine, Stanford University School of Medicine, Stanford, CA, United States of America; 7 Department of Epidemiology, Harvard T.H. Chan School of Public Health, Boston, MA, United States of America; United States Environmental Protection Agency, UNITED STATES

## Abstract

The *Ending the HIV Epidemic* (EHE) Initiative targets a subset of United States (US) priority jurisdictions hardest hit by HIV. It remains unclear which emergency departments (EDs) are the most appropriate targets for EHE-related efforts. To explore this, we used the 2001–2019 National Emergency Department Inventories (NEDI)-USA as a framework to characterize all US EDs, focusing on those in priority jurisdictions and those affiliated with a teaching hospital. We then incorporate multivariable regression to explore the association between ED characteristics and location in an HIV priority jurisdiction. Further, to provide context on the communities these EDs serve, demographic and socioeconomic information and sexually transmitted infection case rate data were included. This reflected 2019 US Census Bureau data on age, race, ethnicity, and proportion uninsured and living in poverty along with 2001–2019 Centers for Disease Control and Prevention case rate data on chlamydia, gonorrhea, and syphilis. We found that EDs in priority jurisdictions (compared to EDs not in priority jurisdictions) more often served populations emphasized in HIV-related efforts (i.e., Black or African American or Hispanic or Latino populations), communities with higher proportions uninsured and living in poverty, and counties with higher rates of chlamydia, gonorrhea, and syphilis. Further, of the groups studied, EDs with teaching hospital affiliations had the highest visit volumes and had steady visit volume growth. In regression, ED annual visit volume was associated with an increased odds of an ED being located in a priority jurisdiction. Our results suggest that geographically targeted screening for HIV in a subset of US priority jurisdiction EDs with a teaching hospital affiliation could be an efficient means to reach vulnerable populations and reduce the burden of undiagnosed HIV in the US.

## Introduction

A 2019 Department of Health and Human Services HIV-related initiative, the *Ending the HIV Epidemic in the U*.*S*. (EHE) Initiative, focuses on a select group of US counties and states hardest hit by HIV—“priority” jurisdictions [1]. These 57 jurisdictions represent areas accounting for half of new infections and include seven states with a substantial rural burden of HIV [1–3]. In these EHE priority jurisdictions, efforts center on diagnosing HIV as early as possible, treating individuals living with HIV rapidly, preventing new HIV transmission, and responding quickly to potential HIV outbreaks [2].

Emergency departments (EDs) routinely serve individuals at higher risk for HIV, e.g., persons engaging in injection substance use presenting after an overdose; EDs are also often safety nets for those with barriers to alternative forms of care, e.g., persons who are uninsured or living in poverty [4–7]. They also treat individuals with undiagnosed HIV and other sexually transmitted infections (STIs) associated with an increased risk of acquiring HIV [4–9]. As such EDs could play an important role in ending the HIV epidemic. So far, their contribution has been insufficient and not in line with longstanding Centers for Disease Control and Prevention (CDC) recommendations [4, 10–12]. Only a minority of ED patients are tested for HIV and less than a quarter of EDs offer any form of routine HIV screening program [11, 12]. Moreover, already low rates of ED-based HIV testing have been exacerbated by the overcrowding and boarding crises in US EDs and already spread-thin resources further strained by COVID-19; recent data indicate that national rates of HIV testing and diagnosis decreased during the pandemic [9, 13, 14].

We recently characterized all EDs in California along with the demographic and socioeconomic characteristics of both the persons served in these EDs and the communities surrounding them [5]. Stratifying these EDs into four groups by EHE HIV priority jurisdiction designation and teaching hospital affiliation, we compared the characteristics of these groups [5]. Incorporating a stratification by teaching hospital affiliation stemmed from the understanding that teaching hospitals are leaders in health care research and innovation [15–17]. Teaching hospitals, compared to non-teaching hospitals, fulfill several social missions, more frequently treat disadvantaged populations, and offer more advanced clinical capabilities [15–17].

In our prior work we found that there are a group of EDs in EHE HIV priority jurisdictions in California–the group also affiliated with a teaching hospital–that could help reduce the burden of undiagnosed HIV in the state [5]. We found that these EDs are major, growing, sources of healthcare in California and compared to other ED groups more often care for vulnerable populations and CDC emphasized communities known to be at higher risk for undiagnosed HIV, e.g., persons experiencing homelessness, individuals who identify as Black or African American race and those who identify as Hispanic or Latino ethnicity. Given their teaching hospital affiliations, these California EDs are also linked to specialized healthcare organizations with the infrastructure and support systems likely required to successfully diagnose, treat, prevent, and respond to HIV. Among all EDs in California, these EDs might also be the most capable of responding to EHE and recent state legislation geared towards strengthening ED-based HIV screening services [18].

Much of the conversation from key stakeholders regarding ED-based HIV screening has revolved around “who” or “how” to test—but not necessarily “where” to test [19, 20]. The first objective of this work was to expand on our previous study and use a similar four-group stratification schema to describe and compare the characteristics of all US EDs by EHE HIV priority jurisdiction designation and teaching hospital affiliation [5]. The second objective was to describe annual visit volume trends among these US ED groups, focusing particularly on those in priority jurisdictions with teaching hospital affiliations. The third objective was to explore the association between ED characteristics and EHE HIV priority jurisdiction designation. The fourth objective was to provide additional context by describing and comparing the socioeconomic, demographic, and STI characteristics of the communities surrounding these EDs, stratified by priority jurisdiction designation.

Given prior work, we hypothesized that EDs in priority jurisdictions—notably those also with a teaching hospital affiliation–would represent important, growing, sources of healthcare in the US for persons at risk for undiagnosed HIV and populations prioritized by the CDC [5]. Given longstanding low rates of HIV screening exacerbated during the COVID-19 pandemic there is a need to understand which EDs might be best positioned to respond to EHE. We believe that EDs with a teaching hospital affiliation represent efficient targets for state and federal resources geared towards ending the HIV epidemic in the US. As such, the broader objective of this study was to make a case for a geographically targeted screening policy that expands routine HIV screening in EDs in EHE priority jurisdictions affiliated with a teaching hospital.

## Materials and methods

### Data sources

We used the most recently available 2001 to 2019 data from the National Emergency Department Inventory (NEDI)-USA to characterize all US EDs [21]. Similar to prior, we stratified EDs by both EHE HIV priority jurisdiction designation and teaching hospital affiliation into four groups—i.e., EDs in priority jurisdictions stratified by with or without a teaching hospital affiliation [groups one and two] and EDs not in priority jurisdictions stratified by with or without a teaching hospital affiliation [groups three and four] [3, 22]. NEDI-USA is a series of national surveys that contains data (e.g., location, annual visit volume, teaching hospital affiliation, and telehealth capabilities) on all non-federal non-specialty EDs in the US [21]. NEDI-USA is a well-established resource utilized in multiple prior studies characterizing US EDs [21, 23–26]. Federal Information Processing System (FIPS) codes for states and counties were used as a crosswalk to identify EDs as either in or not in an EHE HIV priority jurisdiction. Priority jurisdiction classification followed the EHE’s already-established geographic areas of focus described in extensive detail elsewhere [3]. In line with prior, EDs were classified as teaching if they reported an affiliation with an Association of American Medical College’s Council of Teaching Hospitals and Health Systems (COTH) member [22]. ED address was used to categorize EDs by US Census Division and urban influence [27, 28]. The 9 US Census divisions were New England (Connecticut, Massachusetts, Maine, New Hampshire, Rhode Island, and Vermont), Mid Atlantic (New Jersey, New York, and Pennsylvania), East North Central (Illinois, Indiana, Michigan, Ohio, and Wisconsin), West North Central (Iowa, Kansas, Minnesota, Missouri, North Dakota, Nebraska, and South Dakota), South Atlantic (Washington DC, Delaware, Florida, Georgia, Maryland, North Carolina, South Carolina, Virginia, and West Virginia), East South Central (Alabama, Kentucky, Mississippi, and Tennessee), West South Central (Arkansas, Louisiana, Oklahoma, and Texas), Mountain (Arizona, Colorado, Idaho, Montana, New Mexico, Nevada, Utah, and Wyoming), and Pacific (Alaska, California, Hawaii, Oregon, and Washington) [27]. Urban influence classifications incorporated US Department of Agriculture metropolitan statistical area categories; the 3 classifications were urban (categories 1 and 2), large rural (categories 3, 5, and 8), and small rural (categories 4, 6, 7, and 9–12) [28].

Publicly available US Census Bureau data were used to describe and compare county-level demographic and socioeconomic characteristics of the communities these ED groups serve. Demographic information included 2019 county-level population estimates on age, race, and ethnicity; race and ethnicity are based on self-reported data [29]. Socioeconomic information included county-level estimates on the proportion uninsured and proportion living in poverty for all ages; this reflected 2019 Small Area Health Insurance Estimates and Small Area Income and Poverty Estimates, respectively [30, 31]. For additional context, publicly available CDC surveillance reports were used to compare temporal county-level rates on other common STIs. This included county-level population adjusted (per 100,000) surveillance reports on chlamydia, gonorrhea, and syphilis (both primary and secondary) from 2001 to 2019 [32]. Data were adjusted with year-matched county-level US Census Bureau population estimates. In line with current recommendations from the CDC urging caution surrounding inclusion of 2020 data in trend assessments given COVID-19, 2020 STI data were not included [32]. With this exception, all other data were the most recent available versions. With the exception of NEDI-USA data that were ED-level, all other data were county-level. As such, all data could be stratified by priority jurisdiction designation but only NEDI-USA data allowed for further, within-county, stratification by teaching hospital affiliation.

### Statistical analysis

In initial analyses, data were summarized with descriptive statistics (e.g., counts, proportions, medians with interquartile ranges [IQR], means with 95% confidence intervals [CI], and percent changes). Comparisons of categorical variables were completed with χ^2^ tests and continuous variables were compared with Wilcoxon rank sum tests and Kruskal-Wallis tests. In subsequent analyses, we used logistic regression to generate multivariable models exploring the association between ED characteristics and location in an EHE HIV priority jurisdiction. Given the 2019 launch of EHE, data incorporated were from the 2019 NEDI-USA. The primary outcome of interest was priority jurisdiction status. Given prior work, several factors were included as potential predictors [4, 5, 11, 12]. This included annual ED visit volume (per 10,000 visits), urban influence classification, critical access hospital designation, teaching hospital status, and EM residency program status–i.e., did the ED have (or not have) an affiliated EM residency program. These data were summarized with odds ratios (OR) with 95% CIs.

To account for multiple testing, a p value of <0.001 was considered significant. Missing STI data (i.e., 0.2% for each chlamydia, gonorrhea, and syphilis) were not imputed; only available data were analyzed [32]. Stata 15.1 (Stata Corp, College Station, TX) and R version 4.2.1 (https://www.r-project.org) were used to analyze data and create figures. The map was created in ArcGIS Desktop (version 10.8.0; ESRI, Redlands, CA). The Stanford School of Medicine Institutional Review Board determined that this study met criteria for exemption. Data were collected from May 2022 to December 2022. Further, the study followed the Strengthening the Reporting of Observational studies in Epidemiology (STROBE) guidelines [33].

## Results

**Fig 1:**
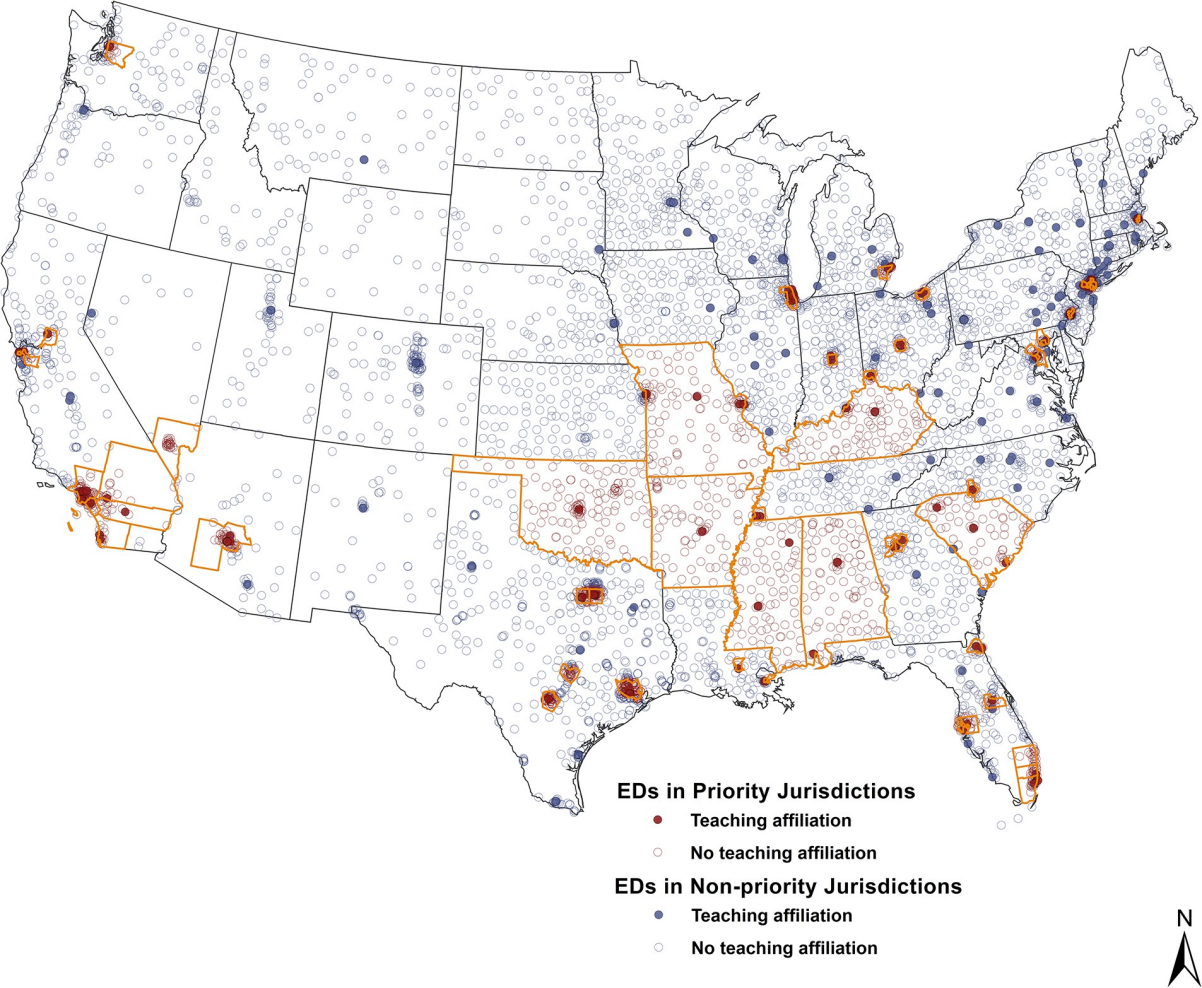
Locations of US emergency departments stratified by priority jurisdiction designation and teaching hospital affiliation. Priority jurisdictions (e.g., counties and states targeted in the Ending the HIV Epidemic in the US initiative) are outlined in gold. Individual ED locations are identified by circles, colored by priority jurisdiction and teaching hospital affiliation status (e.g., those with a teaching hospital affiliation correspond to a solid circle while those without a teaching hospital affiliation correspond to a hollow circle). **Abbreviations**: ED (emergency department).

This analysis included 4,882 (2001) to 5,591 (2019) US EDs that accounted for 101,012,995 to 159,855,260 visits, respectively. In 2019, 31% of all EDs were located in priority jurisdictions. Characteristics of EDs and their surrounding communities stratified by priority designation are presented in **Tables 1** and **S1**, respectively. Characteristics of EDs further stratified by both priority designation and teaching hospital affiliation are presented in **Table 2**. A map showing individual continental US ED location differentiated by priority jurisdiction and teaching hospital affiliation status is presented in **Fig 1**.

**Table 1:** Characteristics of US EDs, stratified by priority jurisdiction designation.

	All Emergency Departments (n = 5,591)		
	EDs in Priority Jurisdiction (n = 1,706)	EDs in Non-Priority Jurisdictions (n = 3,885)	
	n (%)	n (%)	P value
Urban Influence			<0.001
Urban	1,362 (79.8)	2,330 (60.0)	
Large rural	127 (7.4)	645 (16.6)	
Small rural	217 (12.7)	910 (23.4)	
US Census Division			<0.001
New England	10 (0.6)	185 (4.8)	
Mid Atlantic	78 (4.6)	363 (9.3)	
East North Central	135 (7.9)	688 (17.7)	
West North Central	117 (6.9)	575 (14.8)	
South Atlantic	273 (16.0)	584 (15.0)	
East South Central	295 (17.3)	112 (2.9)	
West South Central	507 (29.7)	629 (16.2)	
Mountain	78 (4.6)	419 (10.8)	
Pacific	213 (12.5)	330 (8.5)	
Freestanding ED			<0.001
Satellite ED	205 (12.0)	327 (8.4)	
Autonomous ED	124 (7.3)	152 (3.9)	
Critical Access Hospital	180 (10.6)	1,167 (30.0)	<0.001
Median Visit Volume (IQR)	29,200 (9,855–54,000)	17,000 (6,500–36,500)	<0.001
EM residency program	112 (6.6)	114 (2.9)	<0.001
Teaching hospital	135 (7.9)	128 (3.3)	<0.001
Median number of EDs per county (IQR)	14 (2–48)	2 (1–5)	<0.001

**Abbreviations**: ED (emergency department), IQR (interquartile range)

**Table 2:** Characteristics of US EDs stratified by priority jurisdiction designation and teaching hospital affiliation.

	All Emergency Departments (n = 5,591)				
	EDs in Priority Jurisdictions (n = 1,706)		EDs in Non-Priority Jurisdictions (n = 3,885)		
	Teaching (n = 135)	Not Teaching (n = 1,571)	Teaching (n = 128)	Not Teaching (n = 3,757)	
	n (%)	n (%)	n (%)	n (%)	P value
ED Characteristics					
Urban Influence					<0.001
Urban	134 (99.3)	1,228 (78.2)	126 (98.4)	2,204 (58.7)	
Large rural	1 (0.7)	126 (8.0)	2 (1.6)	643 (17.1)	
Small rural	0 (0)	217 (13.8)	0 (0)	910 (24.2)	
US Census Division					<0.001
New England	6 (4.4)	4 (0.3)	19 (14.8)	166 (4.4)	
Mid Atlantic	19 (14.1)	59 (3.8)	29 (22.7)	334 (8.9)	
East North Central	28 (20.7)	107 (6.8)	20 (15.6)	668 (17.8)	
West North Central	8 (5.9)	109 (6.9)	10 (7.8)	565 (15.0)	
South Atlantic	26 (19.3)	247 (15.7)	25 (19.5)	559 (14.9)	
East South Central	10 (7.4)	285 (18.1)	5 (3.9)	107 (2.9)	
West South Central	15 (11.1)	492 (31.3)	6 (4.7)	623 (16.6)	
Mountain	4 (3.0)	74 (4.7)	9 (7.0)	410 (10.9)	
Pacific	19 (14.1)	194 (12.4)	5 (3.9)	325 (8.7)	
Freestanding ED					<0.001
Satellite ED	0 (0)	205 (13.1)	0 (0)	327 (8.7)	
Autonomous ED	0 (0)	124 (7.9)	0 (0)	152 (4.1)	
Critical Access Hospital	0 (0)	180 (11.5)	0 (0)	1,167 (31.1)	<0.001
Median Visit Volume (IQR)	78,568 (60,000–99,936)	25,550 (9,000–49,000)	74,092 (54,875–95,000)	16,000 (6,200–35,000)	<0.001
EM residency program	67 (49.6)	45 (2.9)	71 (55.5)	43 (1.1)	<0.001
Median number of EDs per county (IQR)	15 (10–48)	14 (2–48)	6 (3–9)	2 (1–5)	<0.001

**Abbreviations**: ED (emergency department), IQR (interquartile range)

Notably, EDs in priority jurisdictions (compared to those not in priority jurisdictions) were more frequently urban, had higher median visit volumes, and were more often affiliated with an emergency medicine residency program and a teaching hospital (all p<0.001, **Table 1**). The counties surrounding these EDs had higher proportions of Black or African American and Hispanic or Latino persons and higher median estimates of proportions uninsured and living in poverty (all p<0.001, **S1 Table**); these counties also had higher–increasing–rates of chlamydia, gonorrhea, and syphilis (**S1 Fig**). EDs affiliated with teaching hospitals (compared to those without a teaching hospital affiliation) in both priority and non-priority jurisdictions were predominately located in urban areas, had the largest median visit volumes, and were more often affiliated with emergency medicine residency programs (all p<0.001 and **Table 2**). Changes in both ED numbers and annual visit volumes over time (2001 versus 2019) are presented in **Table 3**.

**Table 3:** Characteristics of US EDs and visit volumes over time, 2001–2019.

	2001	2019	Change (%)
**Emergency Departments**			
Overall	4,882	5,591	14.5
Priority Jurisdiction	1,424	1,706	19.8
Non-Priority Jurisdictions	3,458	3,885	12.3
Teaching Hospital Affiliation	289	263	-9.0
No Teaching Hospital Affiliation	4,593	5,328	16.0
Priority Jurisdiction and Teaching Hospital Affiliation	158	135	-14.6
**Annual Visit Volumes, in millions**			
Overall	101	160	58.3
Priority Jurisdiction	39	62	60.6
Non-Priority Jurisdictions	62	98	56.8
Teaching Hospital Affiliation	15	21	43.7
No Teaching Hospital Affiliation	86	139	60.7
Priority Jurisdiction and Teaching Hospital Affiliation	8	11	33.0

Emergency department data obtained from the 2001 and 2019 NEDI-USA

ED visit volume trends by year (2001 to 2019) associated with this data are presented in **S2 Fig**. In 2019, visits associated with the 1,706 priority EDs (n = 62,038,496) represented 39% of all ED visits. Visits associated with the 135 priority EDs affiliated with a teaching hospital (n = 10,883,911) represented 7% of all ED visits and 18% of all ED visits in priority jurisdictions. Despite a decrease in the number of EDs with a teaching hospital affiliation (both overall and in priority jurisdictions), steady growth in visit volumes was observed for EDs with a teaching hospital affiliation (44% overall and 33% in priority jurisdictions), although these increases were smaller than the increases for the entire population of US EDs. Output from regression modeling is presented in **Table 4**. In NEDI-USA 2019, three predictors were associated with an ED being located in an HIV priority jurisdiction: annual visit volume, per 10,000 visits (OR 1.06, 95% CI 1.03–1.09), large rural area classification (OR 0.46, 95% CI 0.35–0.59), and critical access hospital designation (OR 0.34, 95% CI 0.27–0.42).

**Table 4:** Association between US ED characteristics and location in an HIV priority jurisdiction.

ED Characteristics, 2019	OR (95% CI)	P value
Annual Visit Volume, per 10,000 Visits	1.06 (1.03–1.09)	<0.001
Urban Influence		
Urban	1.00 (0.81–1.24)	0.99
Large rural	0.46 (0.35–0.59)	<0.001
Small rural	1.00 (reference)	
Critical Access Hospital	0.34 (0.27–0.42)	<0.001
Teaching hospital	1.32 (0.97–1.80)	0.08
EM residency program	1.07 (0.77–1.49)	0.68

**Abbreviations**: CI (confidence interval), ED (emergency department), OR (odds ratio)

## Discussion

Characteristics of ED groups differ by both priority jurisdiction designation and teaching hospital affiliation. EDs in priority jurisdictions (compared to those not in priority jurisdictions) provide a sizable amount of all emergency care in the US. Further, they more often serve those with barriers to care, populations emphasized by EHE and the CDC in HIV-related efforts, and communities more often impacted by STIs beyond just HIV. Within priority jurisdictions, EDs with a teaching hospital affiliation (compared to those without a teaching hospital affiliation) have the largest visit volumes–and visit volumes were associated with the outcome of being located in an HIV priority jurisdiction.

Visit volumes have steadily increased over the preceding years; and so, it seems that the number of visits in all EDs—especially EDs in priority jurisdictions and EDs with teaching hospital affiliations—is anticipated to increase in the coming years. Given recent unwinding of the Medicaid continuous enrollment provision, with an estimated 5 to 14 million persons subsequently losing Medicaid coverage, this will likely represent an increased amount of care being provided to recently uninsured Americans without alternative healthcare options [34]. Given their social mission, EDs in priority jurisdictions affiliated with a teaching hospital will likely represent growing sources of healthcare in the US. Given already low rates of ED-based HIV testing–exacerbated by decreases during the COVID-19 pandemic—this may include an increased number of visits by individuals who are infected with HIV but unaware of their status [13, 14].

Previous ED-based HIV work centers on universal policies without respect to geography. To our knowledge, this work represents the first directed towards understanding “where” (geographically speaking) to test and making a case for focused efforts within EHE’s recently established geographic framework. Much the same as the differing characteristics of ED groups and their surrounding communities described here, rates of HIV are not homogenous across the country [3]. A small number of priority jurisdictions in the US account for the bulk of all new infections in the country–and a small number of EDs provide care to the persons within in these jurisdictions. Further, missed diagnoses most often occur in EDs that most frequently care for at-risk patient populations–EDs in priority jurisdictions with teaching hospital affiliations.

The increased prevalence of HIV in priority jurisdictions suggests that efforts targeted to EDs in these areas might have the highest chance of identifying undiagnosed infections. The unique infrastructure inherent to EDs with a teaching hospital affiliation–along with the clinical and non-clinical specialists crucial to any form of HIV screening and linkage to care program–suggests that efforts further targeted to this subset of EDs might also have the highest likelihood of long-term success. Given the nature and social mission of teaching hospitals in priority jurisdictions, the initiation or expansion of ED-based HIV screening efforts could also spur crucially needed ED-based counseling and pre-exposure prophylaxis (PrEP) programs for at-risk individuals in these EDs [35, 36]. This could reflect either active (e.g., PrEP prescribed in the ED) or passive (e.g., patients linked to outpatient PrEP programs and clinics) approaches. Additionally, once in-place, HIV screening programs could also be expanded to address other ongoing STI epidemics given the growing burden of cases within priority jurisdictions we demonstrate here.

The stated goal of this current study was to make a case for a geographically targeted HIV screening strategy in a group of US EDs. We hoped to do so by further characterizing HIV priority jurisdictions and demonstrating that where, geographically, to test is an important consideration. Our findings offer context for those tasked with determining where best to allocate resources to support EDs responding to EHE. Given finite resources further strained by COVID-19—and the anticipation of increased costs associated with initiating or expanding HIV screening services within EDs affiliated with teaching hospitals—this also includes a renewed call for counties, states, and national organizations to allocate resources into ED settings to help support this mission [37]. In the absence of additional resources and support, cost might impede access to HIV screening and linkage to care among people at risk. Indirectly, we also provide preliminary data suggesting that the CDC’s HIV priority jurisdiction designations are useful for STI-related efforts beyond HIV [3]. Not all EDs serve populations with high rates of HIV and not all EDs have the social mission, infrastructure, or resources to sustain routine HIV screening. However, persistently low rates of ED-based HIV screening suggest that any efforts capable of initiating, or expanding, programs in priority jurisdiction EDs would still be beneficial. Such efforts would likely lead to increased detection of previously undiagnosed infections, reduction in ongoing transmission, earlier linkage to care, and improved outcomes for persons living with HIV.

### Limitations

Our work has multiple limitations and should be interpreted accordingly. First, we acknowledge the limitations associated with the descriptors of Black race and Hispanic or Latino ethnicity for the CDC-emphasized populations highlighted in this work. These are US Census Bureau designations. In line with updated guidance on the reporting of race and ethnicity, we also report county-level socioeconomic indicators (e.g., uninsured status and proportion living in poverty) [38]. Second, given lack of data, we are unable to comment on other communities with higher than average rates of HIV (e.g., men who have sex with men and persons who identify as transgender). Third, we are importantly unable to identify which EDs offer HIV screening services, either routine opt-in or opt-out approaches, or the extent of such testing. To our knowledge, and despite the importance of such information, this data does not exist. Current efforts by our group are aimed at better understanding which US EDs do (or do not) offer routine HIV screening and the underlying reasons why (or why not). Fourth, in line with our prior work, we use teaching hospital affiliation as a proxy for more-resourced hospitals that are more likely to have the infrastructure in place to support ED-based screening services and the specialist physicians and support staff (e.g., infectious disease physicians, counselors and care navigators, and social work services) required to ensure linkage to care and management of newly diagnosed HIV infections. Although NEDI-USA is the most comprehensive national resource on US EDs, we are unable to identify the specific HIV-related resources available in individual EDs. In line with above, our group is currently focused on answering this question. Despite this limitation, our approach remains pragmatic. It is supported by findings from prior work and based in the knowledge that teaching hospitals are uniquely resourced tertiary and quaternary care centers that pioneer advances in medical care, fulfill critical social missions, and train the future physician workforce.

## Conclusions

We demonstrate that there are a group of EDs in priority jurisdictions that, compared to EDs not in priority jurisdictions, serve a population of poorer and uninsured Americans with barriers to care. This ED group also more often cares for populations prioritized by the CDC in HIV-related efforts and communities more often impacted by multiple STIs beyond just HIV. A subset of these priority jurisdiction EDs are affiliated with teaching hospitals that have a social mission to care for marginalized populations. All ED groups studied here reflect growing sources of healthcare in the US.

These findings have immediate health policy implications given recent decreases in HIV testing rates surrounding the COVID-19 pandemic and EHE’s incorporation of a novel geographic framework. As demonstrated by the inadequacy of past efforts, a redoubling of universal ED-based efforts lacking a geographic focus is likely neither feasible nor efficient. Instead, efforts focused on the subset of EDs in priority jurisdictions–namely those also with a teaching hospital affiliation—might be the most efficient, pragmatic, step forward.

## Supporting information

S1 Table Demographic and socieconoimic characteristics, stratified by priority jurisdiction designation. Abbreviations: IQR (interquartile range).
(PDF)

S1 Fig Trends in sexually transmitted infection case rates, stratified by priority jurisdiction designation. Chlamydia, Gonorrhea, and Syphilis (Primary and Secondary) case rate data are limited to 2001–2019; missing data included Chlamydia (n = 133, 0.2%), Gonorrhea (n = 133, 0.2%), and Syphilis (n = 133, 0.2%). Data are stratified by priority (gold) and non-priority (blue) jurisdictions. Mean rates with corresponding 95% CI reflect a year-matched population adjustment (per 100,000 population) and are inclusive all age groups, all race, ethnicities, and both sexes. Only available data were analyzed. *Abbreviations*: *CI (confidence interval)*.
(TIF)

S2 Fig Trends in US emergency department visit volumes over time, stratified by priority jurisdiction designation. Visit volumes trends for EDs in priority jurisdictions (solid gray line) increased from 38,633,600 (2001) to 62,038,496 (2019) visits; over the same period, visit volume ranged from 14,573,804 to 20,949,499 for EDs with a teaching hospital affiliation (dashed blue line) and from 8,184,711 to 10,883,911 for EDs in priority jurisdictions with teaching hospital affiliations (dashed gold line). NEDI-USA was originally conducted every other year and later transitioned to being conducted annually. Years reflect 2001, 2003, 2005, 2007, 2009, 2011, 2012, 2013, and 2015–2019 NEDI-USA emergency department data with a 4/11/22 data cut. *Abbreviations*: *ED (emergency department)*, *NEDI-USA (National Emergency Department Inventory-USA)*, *US (United States)*.
(TIF)

## References

[1] Fauci AS, Redfield RR, Sigounas G, Weahkee MD, Giroir BP. Ending the HIV Epidemic: A Plan for the United States. JAMA. 2019;321(9):844–845. doi: 10.1001/jama.2019.1343 30730529

[2] Ending the HIV Epidemic in the U.S. Published June 7, 2022. Accessed May 15, 2023. https://www.cdc.gov/endhiv/index.html

[3] Jurisdictions. Published June 13, 2022. Accessed May 15, 2023. https://www.cdc.gov/endhiv/jurisdictions.html

[4] Clay CE, Ling A, Bennett CL. HIV Testing at Visits to United States Emergency Departments, 2018. J Acquir Immune Defic Syndr. Published online February 28, 2022. doi: 10.1097/QAI.0000000000002945 35234735 PMC9203905

[5] Bennett CL, Clay CE, Siddiqi KA, Olatosi BA, Parsonnet J, Camargo JCA. Characteristics of California Emergency Departments in Centers for Disease Control and Prevention-Designated HIV Priority Counties. J Emerg Med. 2023;64(1):93–102. doi: 10.1016/j.jemermed.2022.10.020 36650074 PMC10208592

[6] Greenwood-Ericksen MB, Kocher K. Trends in Emergency Department Use by Rural and Urban Populations in the United States. JAMA Netw Open. 2019;2(4):e191919. doi: 10.1001/jamanetworkopen.2019.1919 30977849 PMC6481434

[7] Haukoos JS, Hopkins E, Conroy AA, et al. Routine opt-out rapid HIV screening and detection of HIV infection in emergency department patients. JAMA. 2010;304(3):284–292. doi: 10.1001/jama.2010.953 20639562

[8] White DAE, Giordano TP, Pasalar S, et al. Acute HIV Discovered During Routine HIV Screening With HIV Antigen-Antibody Combination Tests in 9 US Emergency Departments. Ann Emerg Med. 2018;72(1):29–40.e2. doi: 10.1016/j.annemergmed.2017.11.027 29310870

[9] Stanford KA, McNulty MC, Schmitt JR, et al. Incorporating HIV Screening With COVID-19 Testing in an Urban Emergency Department During the Pandemic. JAMA Intern Med. 2021;181(7):1001–1003. 33843944 10.1001/jamainternmed.2021.0839 PMC8042563

[10] Hoover KW, Huang YLA, Tanner ML, et al. HIV Testing Trends at Visits to Physician Offices, Community Health Centers, and Emergency Departments—United States, 2009–2017. MMWR Morb Mortal Wkly Rep. 2020;69(25):776–780. doi: 10.15585/mmwr.mm6925a2 32584800 PMC7316314

[11] Delgado MK, Acosta CD, Ginde AA, et al. National survey of preventive health services in US emergency departments. Ann Emerg Med. 2011;57(2):104–108.e2. doi: 10.1016/j.annemergmed.2010.07.015 20889237 PMC3538034

[12] Berg LJ, Delgado MK, Ginde AA, Montoy JC, Bendavid E, Camargo CA Jr. Characteristics of U.S. emergency departments that offer routine human immunodeficiency virus screening. Acad Emerg Med. 2012;19(8):894–900. doi: 10.1111/j.1553-2712.2012.01401.x 22849642

[13] DiNenno EA, Delaney KP, Pitasi MA, et al. HIV testing before and during the COVID-19 pandemic—United States, 2019–2020. MMWR Morb Mortal Wkly Rep. 2022;71(25):820–824. doi: 10.15585/mmwr.mm7125a2 35737573

[14] Hoover KW, Zhu W, Gant ZC, et al. HIV Services and Outcomes During the COVID-19 Pandemic—United States, 2019–2021. MMWR Morb Mortal Wkly Rep. 2022;71(48):1505–1510. doi: 10.15585/mmwr.mm7148a1 36454696 PMC9721149

[15] Shahian DM, Nordberg P, Meyer GS. Contemporary performance of US teaching and nonteaching hospitals. Academic Medicine. Published online 2012. https://journals.lww.com/academicmedicine/FullText/2012/06000/Contemporary_Performance_of_U_S__Teaching_and.13.aspx?casa_token=8qjrym3lpXAAAAAA:l247Qnemx_nbRHqoyYUhd0DHhjI7qiBs-U1SG0p9UEinWKVIGN55B_bltJ8Hyk5oCFIySlIHdChsDtxgJtEpOw 10.1097/ACM.0b013e318253676a 22534588

[16] Burke LG, Frakt AB, Khullar D, Orav EJ, Jha AK. Association Between Teaching Status and Mortality in US Hospitals. JAMA. 2017;317(20):2105–2113. doi: 10.1001/jama.2017.5702 28535236 PMC5815039

[17] Burke LG, Khullar D, Zheng J, Frakt AB, Orav EJ, Jha AK. Comparison of Costs of Care for Medicare Patients Hospitalized in Teaching and Nonteaching Hospitals. JAMA Netw Open. 2019;2(6):e195229. doi: 10.1001/jamanetworkopen.2019.5229 31173121 PMC6563581

[18] Department of Public Health. California Department of Public Health. Accessed May 15, 2023. https://www.cdph.ca.gov/Programs/CID/DOA/Pages/OA_div_EtE.aspx

[19] Escudero DJ, Bahamon M, Panakos P, Hercz D, Seage GR 3rd, Merchant RC. How to best conduct universal HIV screening in emergency departments is far from settled. J Am Coll Emerg Physicians Open. 2021;2(1):e12352. doi: 10.1002/emp2.12352 33491000 PMC7812459

[20] Haukoos JS, Lyons MS, Rothman RE, et al. Comparison of HIV Screening Strategies in the Emergency Department: A Randomized Clinical Trial. JAMA Netw Open. 2021;4(7):e2117763. doi: 10.1001/jamanetworkopen.2021.17763 34309668 PMC8314142

[21] Sullivan AF, Richman IB, Ahn CJ, et al. A profile of US emergency departments in 2001. Ann Emerg Med. 2006;48(6):694–701. doi: 10.1016/j.annemergmed.2006.08.020 17067721

[22] Council of teaching hospitals and health systems (COTH). AAMC. Accessed May 15, 2023. https://www.aamc.org/career-development/affinity-groups/coth

[23] Carr BG, Branas CC, Metlay JP, Sullivan AF, Camargo CA Jr. Access to emergency care in the United States. Ann Emerg Med. 2009;54(2):261–269. doi: 10.1016/j.annemergmed.2008.11.016 19201059 PMC2728684

[24] Herring AA, Ginde AA, Fahimi J, et al. Increasing critical care admissions from U.S. emergency departments, 2001–2009. Crit Care Med. 2013;41(5):1197–1204. doi: 10.1097/CCM.0b013e31827c086f 23591207 PMC3756824

[25] Brova M, Boggs KM, Zachrison KS, et al. Pediatric Telemedicine Use in United States Emergency Departments. Acad Emerg Med. 2018;25(12):1427–1432. doi: 10.1111/acem.13629 30307078 PMC6822676

[26] Boggs KM, Vogel BT, Zachrison KS, et al. An inventory of stroke centers in the United States. J Am Coll Emerg Physicians Open. 2022;3(2):e12673. doi: 10.1002/emp2.12673 35252972 PMC8886184

[27] US Census Bureau. Geographic Levels. Published online February 14, 2019. Accessed May 15, 2023. https://www.census.gov/programs-surveys/economic-census/guidance-geographies/levels.html

[28] Cromartie J. Urban Influence Codes. Accessed May 15, 2023. https://www.ers.usda.gov/data-products/urban-influence-codes/

[29] US Census Bureau. 2019 Population Estimates by Age, Sex, Race and Hispanic Origin. Published online June 25, 2020. Accessed May 15, 2023. https://www.census.gov/newsroom/press-kits/2020/population-estimates-detailed.html

[30] US Census Bureau. Small Area Health Insurance Estimates (SAHIE) Program. Published online August 11, 2022. Accessed May 15, 2023. https://www.census.gov/programs-surveys/sahie.html

[31] US Census Bureau. Small Area Income and Poverty Estimates (SAIPE) Program. Published online December 15, 2022. Accessed May 15, 2023. https://www.census.gov/programs-surveys/saipe.html

[32] AtlasPlus. Published February 22, 2023. Accessed May 15, 2023. https://www.cdc.gov/nchhstp/atlas/index.htm

[33] Vandenbroucke JP, von Elm E, Altman DG, et al. Strengthening the Reporting of Observational Studies in Epidemiology (STROBE): explanation and elaboration. PLoS Med. 2007;4(10):e297. doi: 10.1371/journal.pmed.0040297 17941715 PMC2020496

[34] Tolbert J, Ammula M. 10 things to know about the unwinding of the Medicaid continuous enrollment provision. KFF. Published April 5, 2023. Accessed May 15, 2023. https://www.kff.org/medicaid/issue-brief/10-things-to-know-about-the-unwinding-of-the-medicaid-continuous-enrollment-provision/

[35] Haukoos JS, White DAE, Rowan SE, et al. HIV Risk and Pre-Exposure Prophylaxis Eligibility Among Emergency Department Patients. AIDS Patient Care STDS. 2021;35(6):211–219. doi: 10.1089/apc.2021.0012 34097464 PMC8336194

[36] Carlisle NA, Booth JS, Rodgers JB, Heath SL, Walter LA. Utilizing Laboratory Results to Identify Emergency Department Patients with Indications for HIV Pre-Exposure Prophylaxis. AIDS Patient Care STDS. 2022;36(8):285–290. doi: 10.1089/apc.2022.0066 35951447

[37] Kelen GD, Wolfe R, D’Onofrio G, et al. Emergency department crowding: the canary in the health care system. NEJM Catalyst Innovations in Care Delivery. 2021;2(5). https://catalyst.nejm.org/doi/abs/10.1056/CAT.21.0217

[38] Flanagin A, Frey T, Christiansen SL, AMA Manual of Style Committee. Updated Guidance on the Reporting of Race and Ethnicity in Medical and Science Journals. JAMA. 2021;326(7):621–627. 34402850 10.1001/jama.2021.13304

