# Supplementary material for: Comparison of US emergency departments by HIV priority jurisdiction designation: A case for geographically targeted screening in teaching hospitals
Source: PLoS One. 2023 Oct 18;18(10):e0292869. doi: 10.1371/journal.pone.0292869 (PMC10584186; doi:10.1371/journal.pone.0292869)
Supplement: S1 Table — Abbreviations: IQR (interquartile range). (PDF) [file pone.0292869.s001.pdf]

S1 Table. Demographic and Socioeconomic Characteristics, Stratified by Priority Jurisdiction Designation

	Priority Jurisdictions, in millions (n=121), n (%)	Non-Priority Jurisdictions, in millions (n=207), n (%)	P value
Hispanic or Latino Ethnicity	30 (24.7)	31 (14.8)	<0.001
Black or African American Race	24 (19.5)	20 (9.9)	<0.001
Age Categories			<0.001
10 to 19	15 (12.5)	27 (12.9)	
20 to 29	18 (14.4)	28 (13.4)	
30 to 39	18 (14.5)	27 (12.9)	
40 to 49	15 (12.6)	25 (12.1)	
50 to 59	15 (12.6)	27 (13.1)	
60 to 69	13 (10.8)	25 (12.1)	
Median Estimates of Uninsured for All Ages, % (IQR)	15.6 (11.3-19.4)	12.1 (8.8-17.1)	<0.001
Median Estimates of Poverty for All Ages, % (IQR)	16.3 (13.6-19.8)	12.0 (9.4-15.3)	<0.001

Abbreviations: IQR (interquartile range)
