# Supplementary figures and images for: Comparison of US emergency departments by HIV priority jurisdiction designation: A case for geographically targeted screening in teaching hospitals
Source: PLoS One. 2023 Oct 18;18(10):e0292869. doi: 10.1371/journal.pone.0292869 (PMC10584186; doi:10.1371/journal.pone.0292869)

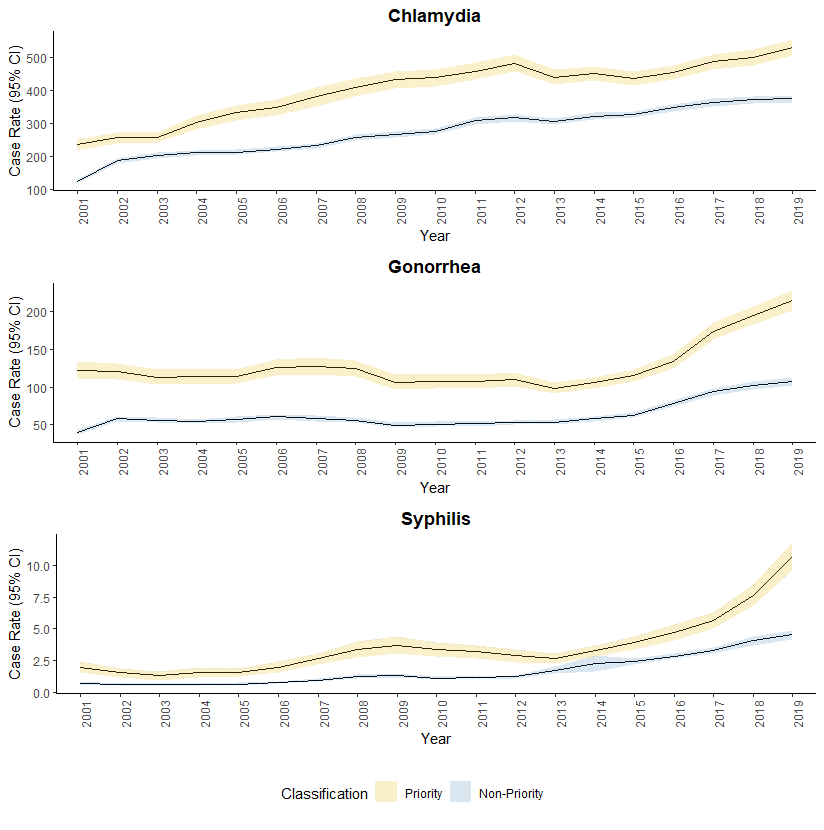

Supplement: S1 Fig — Chlamydia, Gonorrhea, and Syphilis (Primary and Secondary) case rate data are limited to 2001–2019; missing data included Chlamydia (n = 133, 0.2%), Gonorrhea (n = 133, 0.2%), and Syphilis (n = 133, 0.2%). Data are stratified by priority (gold) and non-priority (blue) jurisdictions. Mean rates with corresponding 95% CI reflect a year-matched population adjustment (per 100,000 population) and are inclusive all age groups, all race, ethnicities, and both sexes. Only available data were analyzed. Abbreviations: CI (confidence interval). (TIF) [file pone.0292869.s002.tif]

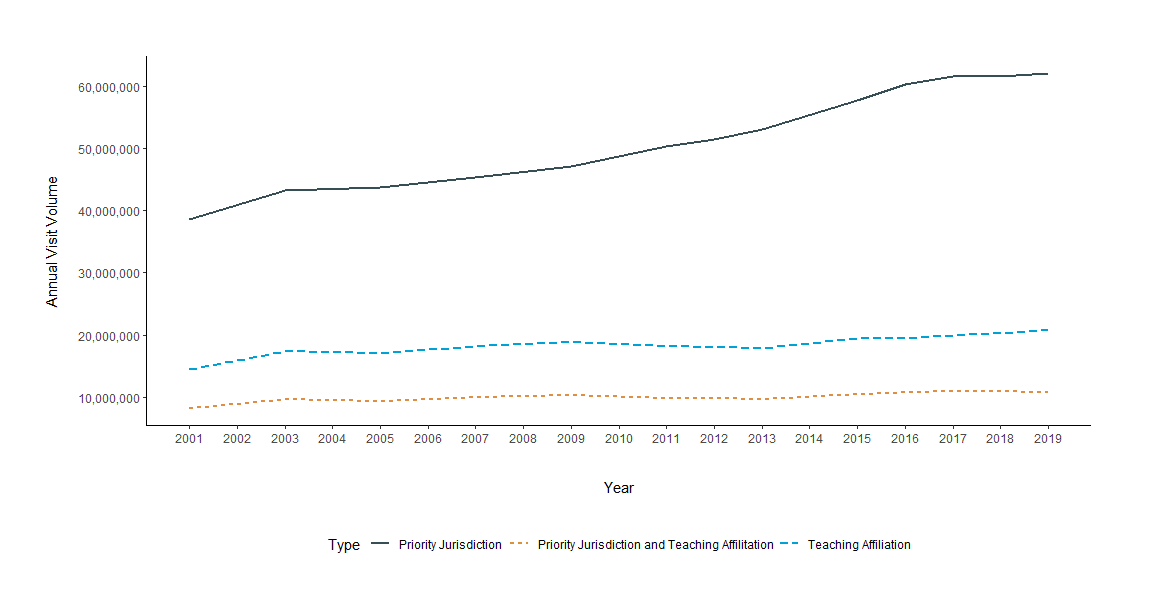

Supplement: S2 Fig — Visit volumes trends for EDs in priority jurisdictions (solid gray line) increased from 38,633,600 (2001) to 62,038,496 (2019) visits; over the same period, visit volume ranged from 14,573,804 to 20,949,499 for EDs with a teaching hospital affiliation (dashed blue line) and from 8,184,711 to 10,883,911 for EDs in priority jurisdictions with teaching hospital affiliations (dashed gold line). NEDI-USA was originally conducted every other year and later transitioned to being conducted annually. Years reflect 2001, 2003, 2005, 2007, 2009, 2011, 2012, 2013, and 2015–2019 NEDI-USA emergency department data with a 4/11/22 data cut. Abbreviations: ED (emergency department), NEDI-USA (National Emergency Department Inventory-USA), US (United States). (TIF) [file pone.0292869.s003.tif]
